# Molecular characterization of antibiotic resistance in bacteria from daycare centres in Ile-Ife, Nigeria

**DOI:** 10.1093/jacamr/dlae213

**Published:** 2024-12-30

**Authors:** Eunice Damilola Wilkie, Jude Oluwapelumi Alao, Oluwakemi Abike Thonda, Anthonia Olufunke Oluduro

**Affiliations:** Microbiology Department, Adeleke University, Ede, Osun, Nigeria; School of Public Health and Interdisciplinary Studies, Auckland University of Technology, Auckland, New Zealand; Department of Microbiology, Babcock University, Ilishan-Remo, Ogun State, Nigeria; Department of Microbiology, Obafemi Awolowo University, Ife, Osun, Nigeria

## Abstract

**Background:**

Antibiotic resistance is an escalating global health issue, with particularly severe implications in low- and middle-income countries (LMICs) such as Nigeria. This study examines antibiotic-resistant bacteria’s prevalence and molecular characteristics in daycare centres in Ile-Ife, Nigeria, where high antibiotic use and limited infection control measures present significant challenges.

**Methods:**

Between November 2017 and July 2019, samples were collected from 20 daycare centres, including swabs from fomites and children. Bacterial isolates were identified and assessed for antibiotic susceptibility using standard methods. Molecular techniques, including PCR, were employed to detect resistance genes such as *blaSHV*, *tetA*, *dfr1* and *mecA*.

**Results:**

The study found high resistance levels among common pathogens, with *S. aureus* and other staphylococci showing significant resistance to ampicillin and Augmentin and Gram-negative bacteria exhibiting broad resistance patterns. Resistance genes, including *blaSHV* and *mecA*, were identified in multiple isolates, indicating the spread of crucial resistance mechanisms.

**Conclusions:**

The results highlight the critical need for improved surveillance, targeted antimicrobial stewardship and enhanced infection control practices in daycare centres to address the growing threat of antibiotic resistance. This research offers valuable insights into resistance dynamics in paediatric settings and supports the development of strategies to manage the spread of resistant bacteria in LMIC contexts.

## Introduction

Antibiotic resistance has emerged as one of the most critical global health challenges of the 21st century, with profound implications for public health and economic stability. Inadequate infection control measures and the overuse of antibiotics have led to the proliferation of resistant bacteria, rendering many standard treatments ineffective and resulting in prolonged infections, increased healthcare costs and higher mortality rates.^1,2^ The WHO has underscored the urgency of addressing antibiotic resistance, emphasizing the need for coordinated global action.^3^

In low- and middle-income countries (LMICs), such as Nigeria, the problem of antibiotic resistance is particularly acute. The healthcare infrastructure in these regions often struggles with insufficient resources, leading to widespread misuse of antibiotics and inadequate infection control practices.^4^ This exacerbates the problem of antibiotic resistance, making it a pressing concern in these settings.

Daycare centres, where young children interact closely, represent a critical environment for the spread of infectious agents, including antibiotic-resistant bacteria. Young children are especially vulnerable due to their developing immune systems and high frequency of hand-to-mouth activities, which increase their exposure to pathogens.^5^ Furthermore, the high usage of antibiotics in paediatric care contributes to selective pressure, fostering the emergence and spread of resistant strains.^6^ Despite these challenges, there is a significant gap in understanding the molecular basis of antibiotic resistance in these settings, particularly in LMIC contexts like Ile-Ife, Nigeria.

Ile-Ife, a city in south-western Nigeria, exemplifies these challenges with its daycare centres potentially serving as hotspots for transmitting resistant bacteria. The current surveillance and understanding of antibiotic resistance in these settings are limited, leaving a crucial knowledge gap regarding the specific resistance genes and their prevalence.

This study aims to address this gap by molecularly identifying and characterizing antibiotic-resistant genes in bacterial isolates from daycare centres in Ile-Ife. Utilizing advanced molecular techniques, we seek to determine the prevalence and diversity of resistance genes and elucidate the mechanisms driving resistance in this specific context. The insights gained from this study will enhance our understanding of antibiotic resistance dynamics in paediatric environments within LMICs and provide valuable information for developing targeted interventions to control the spread of resistant bacteria in daycare centres and similar settings.

## Materials and methods

### Ethics

The study was conducted following the approval from the Health Research Ethics Committee (HREC) at the Institute of Public Health, Obafemi Awolowo University, Ile-Ife, Nigeria (HREC Number: IPHOAU/12/1337). Informed consent was secured from the parents of the children attending the daycare centres and daycare centre workers prior to sample collection. Data were anonymized to protect the confidentiality of the participants and the centres involved.

### Sample collection

The study was conducted from November 2017 to July 2019, in Ile-Ife, a historical city in Osun State, Nigeria. Known for its blend of urban and rural areas, Ile-Ife hosts a diverse population, including families engaged in government work, agriculture, trading and artisan activities, offering a blend of socioeconomic backgrounds and lifestyle exposures. Twenty daycare centres were selected across both urban and rural settings to ensure a representative sample. Each centre was visited twice for sample collection from various fomites (toys, diaper changing areas, tables, mats, door handles and bedsheets) and from children’s and daycare workers’ palms, fingers and nostrils. Sterile cotton swabs moistened with sterile saline were used for sample collection.

### Questionnaire administration and data collection

Parents/guardians of the children and daycare workers were interviewed using a structured questionnaire (File S1, available as Supplementary data at *JAC-AMR* Online). The questionnaire gathered socio-demographic information, medical history of the children and occupational details of the parents/guardians and workers. The collected data were analysed to understand the study population’s background comprehensively.

### Inclusion and exclusion criteria

- Inclusion criteria: children aged between 6 and 42 months and daycare workers.
- Exclusion criteria: (i) parents who declined or were unwilling to allow samples to be taken from their children; (ii) children over 48 months old; and (iii) children undergoing antibiotic therapy.

### Bacterial isolation and identification

Upon arrival at the laboratory, swabs were vortexed in 1 mL of sterile phosphate-buffered saline to release the bacteria. A portion of the resulting suspension was cultured on various media, including nutrient agar, eosin methylene blue (EMB) agar, Mueller–Hinton agar, nutrient broth, MacConkey agar, triple sugar iron agar, sulphide indole motility agar, mannitol salt agar and blood agar plates (Oxoid, Himedia). The plates were incubated at 37°C for 24–48 h.

Biochemical tests included catalase test, citrate test, indole test, methyl red and Voges–Proskauer tests, sugar fermentation, motility test, oxidase test, hydrogen sulphide production, DNase test and spore-forming test.

### Antibiotic susceptibility test

The antibiotics chosen for susceptibility testing were selected based on common prescription practices in Nigeria, reflecting those frequently used to treat infections in healthcare settings.^7^ Antibiotic susceptibility was determined using the Kirby–Bauer disk diffusion method on Mueller–Hinton agar plates. For Gram-positive bacteria, antibiotic disks from Biomark Laboratories, India, included gentamicin (10 µg), amoxicillin/clavulanic acid (Augmentin) (30 µg), ceftazidime (30 µg), cephalexin (1.5 µg), cefuroxime (10 µg), erythromycin (5 µg), vancomycin (30 µg), cotrimoxazole (25 µg), ampicillin (10 µg), tetracycline (30 µg), ciprofloxacin (5 µg), cefuroxime (10 µg) and ceftazidime (10 µg). For Gram-negative bacteria, antibiotic disks from Abtek Biological Ltd and Oxoid, England, included gentamicin (10 µg), tetracycline (10 µg), meropenem (10 µg), cotrimoxazole (30 µg), chloramphenicol (30 µg), ciprofloxacin (5 µg), trimethoprim (5 µg), nitrofurantoin (300 µg), ofloxacin (5 µg), Augmentin (30 µg), amoxicillin/clavulanic acid (30 µg) and cefotaxime (30 µg).

### Molecular analysis for the detection of antibiotic resistance in bacterial isolates

#### DNA extraction

Bacterial DNA was extracted by suspending bacterial colonies in 200 µL of sterile distilled water in appropriately labelled Eppendorf tubes. The tubes were sealed with paraffin tape to prevent accidental opening. The bacterial suspensions were then heated at 100°C for 15 min in a water bath, followed by centrifugation at 10 000 rpm for 15 min. The supernatant containing the DNA was used for subsequent PCR studies and stored at 4°C.^8,9^

### PCR procedure for the detection of *Staphylococcus aureus* and *MecA*

The selected targets for PCR, including *S. aureus* and its methicillin-resistance gene *mecA*, were chosen based on their clinical relevance and the prevalence of antibiotic resistance patterns observed in local isolates. *S. aureus* is a major human pathogen, and the emergence of MRSA poses significant challenges in both healthcare and community settings, including daycare environments, which is why it was prioritized for molecular analysis.^10^ The selection of *mecA* was driven by its critical role in conferring resistance to methicillin, a commonly used antibiotic in Nigeria. Primers specific for detecting *S. aureus* and *mecA* were employed, as outlined in File S2. The PCR mixture included 12.5 µL of master mix, 0.5 µL each of forward and reverse primers, 8.5 µL of nuclease-free water and 3 µL of template DNA, bringing the total volume to 25 µL.

The PCRs were performed in a PCR thermocycler (Applied Biosystems, Singapore) under specific conditions for each target. For each run, a 100 bp DNA ladder from Thermo Fisher Scientific was used as the molecular weight DNA standard to verify the proper size of each PCR product. For *S. aureus*, the procedure began with an initial denaturation at 94°C for 5 min, followed by 37 cycles of denaturation at 94°C for 1 min, annealing at 50°C for 30 s and extension at 72°C for 1 min. A final extension step was carried out at 72°C for 5 min.

For *mecA* detection, the PCR process started with an initial denaturation at 95°C for 3 min. This was followed by 35 cycles of denaturation at 94°C for 1 min, annealing at 48°C for 30 s and extension at 72°C for 1 min. The final extension step was completed at 72°C for 6 min.

### PCR procedure for the detection of antibiotic resistance genes

Each bacterium chosen for molecular analysis was selected based on its clinical relevance and potential to harbour antibiotic resistance, as identified during preliminary findings of this study. *Citrobacter youngae*, although less frequently discussed in clinical settings, is relevant due to its increasing association with healthcare-associated infections and its potential to acquire resistance genes.^11^  *Klebsiella oxytoca* and *K. pneumoniae* are both clinically significant pathogens known for their role in respiratory and urinary tract infections and are notorious for acquiring resistance genes, particularly for beta-lactam antibiotics.^12^  *Escherichia coli*, a well-known cause of gastrointestinal and urinary tract infections,^13^ was included due to its high prevalence and role as a major carrier of resistance determinants. *Providencia rettgeri* was analysed because it can cause opportunistic infections and is often associated with multidrug resistance,^14^ particularly in immunocompromised patients.

The selected PCR targets for detecting specific antibiotic-resistant genes (*blaSHV, tetA, aac(3)-II* and dfr1) were based on the prevalent antibiotypes observed among the isolates, which reflect common resistance patterns linked to antibiotic usage in Nigeria. These genes were chosen due to their association with resistance to antibiotics frequently prescribed in local healthcare settings, making them relevant markers for assessing resistance in this context (File S2). The PCR process was carried out with slightly varied conditions tailored to each gene. For the *blaSHV* gene, amplification began with an initial denaturation at 94°C for 5 min, followed by 35 cycles of denaturation at 94°C for 1 min, annealing at 49°C for 1 min and extension at 72°C for 2 min, finishing with a final extension at 72°C for 10 min. Identical conditions were used for the *tetA, aac(3)-II* and *dfr1* genes to ensure consistent amplification.

### Gel electrophoresis

PCR products were separated on a 1.5% agarose gel. This gel was prepared by dissolving 1 g of agarose powder (Cleaver Scientific, UK) in 100 mL of 1× Tris–borate–EDTA (TBE) buffer (Bioconcept, Ltd, Switzerland) in a clean conical flask. The 1.5% agarose solution was heated in a microwave for 2–3 min until clear, indicating complete dissolution. The mixture was then cooled to about 50°C, after which 5 µL of ethidium bromide was added. It was further cooled before being poured into a tray sealed at both ends to form a mould, with special combs placed in it to create wells. Once the gel set, the comb was carefully removed, and the gel plate was placed in an electrophoretic tank containing 1× TBE solution. Five microlitres of the amplicon was mixed with 5 µL of quick-load purple 100 bp DNA ladder (loading buffer). The 10 µL mixture was loaded into the wells of the agarose gel. The power supply was set to 100 volts for 25 min, with the gel immersed in the 1× TBE buffer in the tank.^15^ A 100 base-pair molecular weight DNA standard (size marker) was used to verify the correct size of each PCR product.

The DNA bands were visualized and captured using a short-wave ultraviolet transilluminator and photographed with a gene gel bioimaging system. The PCR products were analysed by comparing the various DNA bands to the 100 bp DNA standard.

## Results

### Baseline characteristics of the study population

The study was conducted between November 2017 and July 2019, involving 233 samples from 76 children, 33 daycare workers and 124 fomites across 17 daycare centres in Ile-Ife. Initially, 159 individuals were approached; however, some parents or daycare workers declined participation and others were excluded due to ongoing antibiotic therapy among the children or daycare workers. Among the enrolled children, 40 (52.63%) were males and 36 (47.37%) were females, aged between 6 and 42 months. Most mothers (53.95%) were public servants, with a significant portion (25%) being farmers, traders or artisans.

### Cultural and morphological characteristics of bacteria

The selection of pathogens, including less commonly associated organisms such as *Corynebacterium youngae* and *Providencia rettgeri*, was based on preliminary findings from the study. These isolates were identified during the initial analysis of samples and were found to represent the diversity of the bacterial community in the sampled daycare centres. While other healthcare-associated pathogens, such as *Enterobacter aerogenes*, *Citrobacter freundii* and *Providencia stuartii*, are more commonly reported in clinical settings, they were not prominently recovered in this study. This reflects the specific microbial profile of the sampled daycare environments rather than general healthcare settings.

*Staphylococcus aureus* isolates were phenotypically observed as yellowish, non-mucoid colonies on mannitol salt agar, with some fermenting mannitol but testing negative for DNase. Other *Staphylococcus* species appeared pinkish on mannitol salt agar. *Escherichia coli* isolates were observed as large, pink-rose colonies with a green metallic sheen on EMB agar.

*Bacillus* sp. was moderate to large, irregular in shape and whitish on nutrient agar. *Pseudomonas aeruginosa* colonies were moderate in size, raised, entire in shape and exhibited a green pigment on nutrient agar. *Corynebacterium kutscheri* and *Corynebacterium xerosis* produced brownish colonies on nutrient agar, raised with entire edges.

Threshold criteria for identification included observable growth on selective media and colony morphology characteristic of the target species.

### Biochemical identification of bacterial isolates

Targeted bacterial isolates were subjected to biochemical tests to confirm their identity. *S. aureus* was positive for DNase, catalase and mannitol fermentation but negative for oxidase. *Bacillus* species, although not part of the initial target pathogens, were identified based on catalase and oxidase positivity and their distinct spore formation. Similarly, *C. xerosis* and *C. kutscheri* were included after identification through their biochemical profiles, including variations in starch hydrolysis and oxidase activity.

Among the Gram-negative bacteria, *E. coli, P. aeruginosa* and *K. pneumoniae* were identified through their distinct biochemical characteristics. Notably, *C. youngae*, a targeted pathogen, was not isolated in the samples analysed. Instead, members of the Enterobacteriaceae, as well as non-target organisms such as *Pseudomonas* species, were identified based on their growth on selective media and confirmed through biochemical testing.

### Overall distribution of bacterial isolates in daycare centres

The distribution of bacterial isolates stratified by source is presented in Table 1, highlighting the recovery of pathogens from fomites, children and daycare workers.

**Table 1.** Frequency and percentage distribution of total bacteria isolated from daycare centres in Ile-Ife

Category	Bacterial isolates	Children (*n*)	Daycare workers (*n*)	Fomites (*n*)	Total (*n*)	Percentage (%)
**Gram-positive bacteria**						
	*Staphylococcus aureus*	8	6	6	20	4.12
	*Staphylococcus* sp.	30	20	24	74	15.25
	*Bacillus* sp.	100	75	75	250	51.55
	*Corynebacterium xerosis*	25	20	18	63	12.98
	*Corynebacterium kutscheri*	15	12	18	45	9.28
**Gram-negative bacteria**						
	*Pseudomonas aeruginosa*	1	1	0	2	0.41
	*E. cloacae*	0	0	1	1	0.21
	*E. agglomerans*	1	0	2	3	0.62
	*P. rettgeri*	1	1	2	4	0.83
	*P. stuartii*	0	1	0	1	0.21
	*Proteus* sp.	0	1	0	1	0.21
	*P. mirabilis*	1	0	0	1	0.21
	*K. pneumoniae*	1	1	0	2	0.41
	*K. oxytoca*	1	2	2	5	1.03
	*Klebsiella* sp.	0	0	1	1	0.21
	*E. coli*	1	1	0	2	0.41
	*Serratia* sp.	1	0	1	2	0.41
	*Serratia liquefaciens*	0	1	0	1	0.21
	*Enterobacter* sp.	1	0	2	3	0.62
	*C. youngae*	0	0	1	1	0.21
	Others	0	0	2	2	0.41
**Total**		**186**	**142**	**157**	**485**	**100%**

### Antibiotic susceptibility profile

The antibiotic susceptibility profiles of bacterial isolates are summarized in Table 2, with specific focus on the number of isolates in each group for context.

**Table 2.** Antibiotic susceptibility profile of bacterial isolates

Organism	Subgroup	Number of isolates	Susceptible antibiotics	Resistant antibiotics
*Staphylococcus aureus*	Gram-positive cocci	14	Gentamicin (100%), Ciprofloxacin (100%)	Ampicillin (85.7%), Augmentin (85.7%)
*Staphylococcus* sp.	Gram-positive cocci	9	Gentamicin (94.44%), Ciprofloxacin (100%)	Ampicillin (88.89%), Augmentin (83.3%)
*Bacillus* sp.	Gram-positive bacilli	27	Gentamicin (94.44%), Ciprofloxacin (94.44%)	Cefuroxime (98.15%), Ampicillin (98.15%)
*Corynebacterium* sp.	Gram-positive bacilli	24	Ciprofloxacin (85.41%), Gentamicin (79.17%)	Cefuroxime (91.67%), Ampicillin (95.83%)
Enterobacteriaceae	Gram-negative bacilli (combined)	16	Augmentin (100%), Cefotaxime (100%)	Trimethoprim (40%), Tetracycline (20%)
*Pseudomonas aeruginosa*	Gram-negative bacilli	4	Gentamicin (100%), Ciprofloxacin (100%)	Chloramphenicol (50%), Tetracycline (50%)

Significant differences (*P* < 0.05) were observed in susceptibility and resistance patterns among the bacterial isolates, particularly in Gram-negative organisms.

### Molecular detection of resistance genes

**Figure 1.**
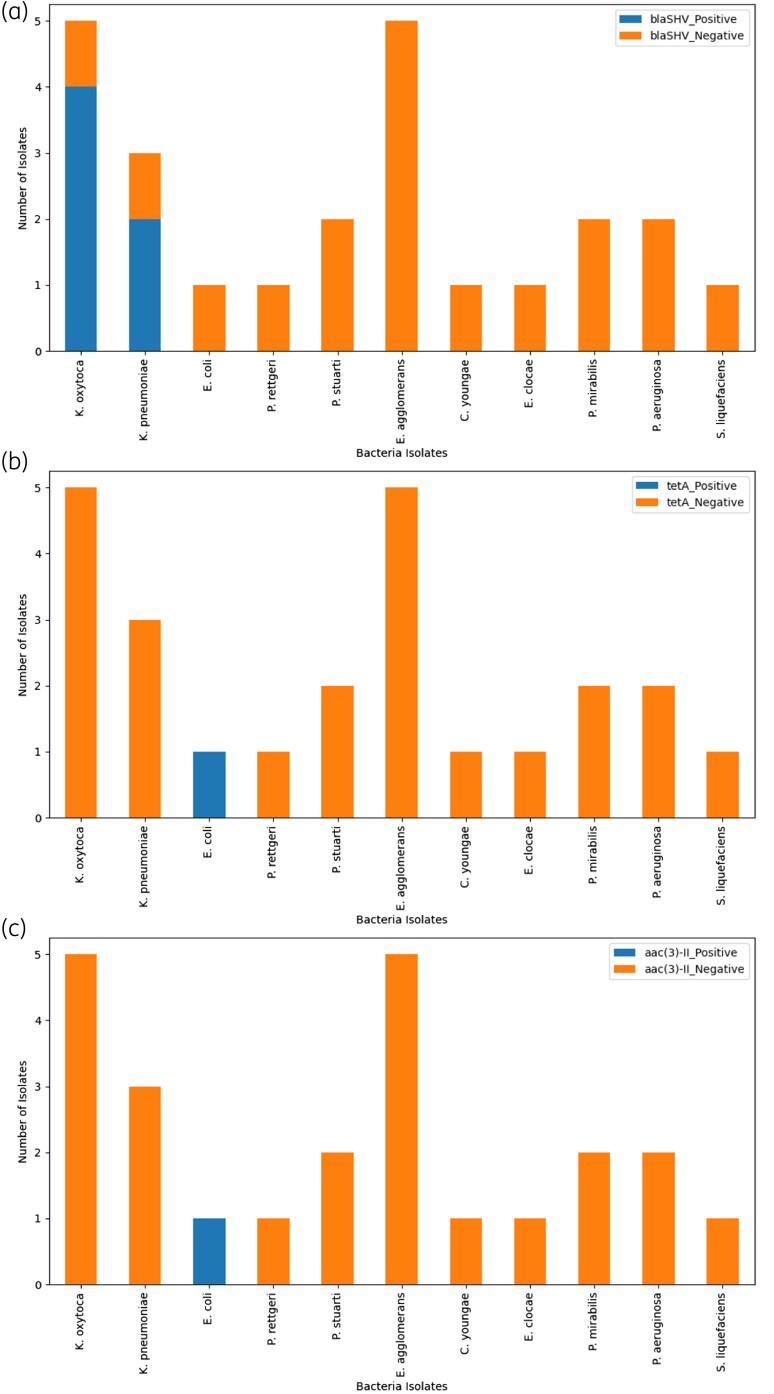
Frequency and percentage distribution of resistance genes in Gram-negative bacteria. (a) Distribution of blaSHV gene in Gram-negative bacteria. (b) Distribution of tetA gene in Gram-negative bacteria. Distribution of aac(3)-II gene in Gram-negative bacteria.

The molecular detection results for resistance genes in Gram-negative bacteria are summarized in Table 3. The distribution of these genes is visually presented in Figure 1, and the gel blots are shown in File S3.

**Table 3.** Molecular detection of resistance genes in Gram-negative bacteria

Resistance gene	Target organisms	Number of isolates tested	Number of positives	Detection rate (%)
*blaSHV*	*Klebsiella pneumoniae*, *Providencia rettgeri*	24	7	29.2
*tetA*	*Escherichia coli*	24	1	4.2
*dfr1*	*Enterobacteriaceae*	32	7	21.9
*aac(3)-II*	*Pseudomonas aeruginosa*	24	1	4.2
*mecA*	*Staphylococcus aureus*	20	2	16.67

## Discussion

The findings from this study align with similar research conducted both in Nigeria and internationally, highlighting the pervasive issue of antibiotic resistance in diverse settings. Several studies conducted in Nigeria on samples from children in varied environments have reported similar bacterial species and high levels of antibiotic resistance.^16–18^ This mirrors the high resistance rates observed in this study, emphasizing the widespread nature of the problem across the country. These results underscore the need for robust interventions targeting the drivers of resistance at community levels, particularly in daycare centres where young children are especially vulnerable to infections. Similarly, Chukwu *et al*.^19^ assessed public awareness of AMR in Nigeria, finding a general public lack of awareness and knowledge about AMR. This lack of awareness not only fosters the misuse of antibiotics but also hampers community-level efforts to mitigate the spread of resistant pathogens. Daycare centres, as microcosms of community interaction, provide a unique opportunity to implement targeted educational campaigns aimed at both caregivers and parents. Such interventions could complement broader national efforts to combat AMR and mitigate its public health impact.

The broader context of sub-Saharan Africa also shows significant antibiotic resistance challenges. Studies across the region have consistently reported high levels of resistance in community settings, often driven by similar factors such as the overuse and misuse of antibiotics, poor infection control practices and inadequate public health infrastructure. For instance, research in Kenya and South Africa has highlighted the presence of multidrug-resistant bacteria in both healthcare and community environments,^20–22^ drawing parallels to the findings of this study. The resistance patterns observed in this study are not isolated but part of a larger global trend, emphasizing the universal challenge AMR poses. Genomic characterization studies, such as those by Uwanibe *et al*.^23^, have identified numerous resistance and virulence genes in multidrug-resistant bacteria in Nigeria, including *blaSHV*, *tetA, dfr1* and *mecA* genes. The detection of these genes in this study’s isolates aligns with previous findings, suggesting that genetic determinants of resistance are widespread and demand urgent attention for their potential in facilitating horizontal gene transfer between pathogens.

One notable finding is that only 65% (13 out of 20) of isolates initially identified as *S. aureus* based on phenotypic methods were confirmed using 16S rRNA analysis. This discrepancy highlights the limitations of relying solely on phenotypic identification methods, which may lead to misidentification, particularly in cases where non-*S. aureus* staphylococci or other Gram-positive cocci exhibit similar morphological characteristics. The remaining 35% of unconfirmed isolates could potentially represent coagulase-negative *Staphylococcus* species, such as *S. epidermidis*, which is known to be part of the skin flora but may occasionally exhibit similar resistance profiles.^24^ This underscores the necessity of molecular confirmation for accurate bacterial identification, particularly in studies focusing on antibiotic resistance.

The high levels of antibiotic resistance observed in this study have significant public health implications, particularly in LMICs like Nigeria and other parts of sub-Saharan Africa. High resistance rates to commonly prescribed antibiotics such as ampicillin and Augmentin in *S. aureus*, other staphylococci and *Bacillus* species pose a major threat to effective infection management in daycare centres. The high resistance rates to commonly prescribed antibiotics such as ampicillin and Augmentin observed in *S. aureus*, other staphylococci and *Bacillus* species highlight a concerning trend of reduced effectiveness of these treatments, posing a threat to effective infection management in daycare centres. Specifically, *S. aureus* showed resistance to ampicillin (85.7%) and Augmentin (85.7%), while other staphylococci exhibited similar resistance levels to ampicillin (88.89%) and Augmentin (83.3%). These findings are particularly concerning in daycare settings, where children are in close contact with one another and with contaminated fomites, creating an environment conducive to the transmission of antibiotic-resistant pathogens.

Additionally, the resistance of Enterobacteriaceae to trimethoprim and tetracycline, along with *P. aeruginosa’*s resistance to chloramphenicol and tetracycline, reflects global trends of multidrug resistance in Gram-negative bacteria. These findings highlight the urgent need for enhanced hygiene practices in daycare centres to limit the spread of these pathogens. Without such interventions, the risk of outbreaks caused by multidrug-resistant bacteria remains high, threatening the health and well-being of children.

Robust surveillance systems are critical to regularly monitoring antibiotic resistance patterns in daycare centres. Such systems can facilitate early detection of emerging resistance trends, enabling timely and effective public health interventions. Additionally, implementing antimicrobial stewardship programmes tailored for paediatric care settings is crucial. These programmes should prioritize the rational use of antibiotics, providing education for healthcare providers on best practices and involving parents and caregivers in efforts to reduce unnecessary antibiotic use.

Improved infection control measures in daycare centres are also essential. These measures should include rigorous hand hygiene protocols, regular cleaning and disinfection of toys and surfaces and the use of personal protective equipment where appropriate. Training daycare staff on infection prevention and control practices can significantly reduce the transmission of resistant pathogens. Furthermore, public health campaigns to raise awareness about the dangers of antibiotic resistance and the importance of appropriate antibiotic use are crucial. These campaigns should focus on educating parents, caregivers and the wider community, fostering a culture of informed and responsible antibiotic consumption.

Several limitations must be acknowledged. First, the sample size of 233 isolates may not fully capture the diversity of bacterial flora or the extent of antibiotic resistance in daycare centres in the region. Second, the reliance on phenotypic methods for initial identification may have contributed to misidentification, as indicated by the discrepancy in *S. aureus* identification rates. Third, the study’s observational nature limits its ability to establish causative relationships between community antibiotic use and observed resistance patterns.

Furthermore, while the detection of resistance genes such as *blaSHV, tetA, dfr1* and *mecA* provides valuable insights into the genetic mechanisms underlying resistance, this study did not perform whole-genome sequencing, which could have offered a more comprehensive view of the resistome and its potential for horizontal gene transfer. Future studies employing advanced molecular techniques would be beneficial for a more detailed genetic characterization of the isolates.

### Conclusion

The study highlights the significant challenge antibiotic-resistant bacteria pose in daycare centres in Ile-Ife, Nigeria. The high prevalence of resistance and detection of critical resistance genes underscores the urgent need for coordinated efforts to combat this public health threat. Implementing targeted interventions, enhancing public awareness and fostering a comprehensive understanding of resistance dynamics can help mitigate the spread of antibiotic-resistant bacteria. The findings contribute to a broader understanding of antibiotic resistance in paediatric environments and inform strategies to curb the spread of resistant bacteria, ultimately protecting public health on a larger scale.

## Supplementary Material

dlae213_Supplementary_Data

## Data Availability

All data associated with this study are provided in the manuscript.

## Supplementary data

Files S1 to S3 are available as Supplementary data at *JAC-AMR* Online.
