# Supplementary material for: Molecular characterization of antibiotic resistance in bacteria from daycare centres in Ile-Ife, Nigeria
Source: JAC Antimicrob Resist. 2024 Dec 30;7(1):dlae213. doi: 10.1093/jacamr/dlae213 (PMC11683008; doi:10.1093/jacamr/dlae213)
Supplement: dlae213_Supplementary_Data [file dlae213_supplementary_data.zip › Supplementary File 2.docx]

**Primers for the Detection of *S. aureus***

| **Gene** | **Primer Sequence (5'-3')** | **Size (bp)** | **Reference** |
| --- | --- | --- | --- |
| *STPY* | F: ACGGTCTTGCTGTCACTTATA | 257 | [1] |
|  | R: TACACATATGTTCTTCCCTAATAA |  |  |

**Primers for the Detection of *mecA* in *S. aureus* Isolates**

| **Gene** | **Primer Sequence (5'-3')** | **Size (bp)** | **Reference** |
| --- | --- | --- | --- |
| *mecA* | F: GCA ATC GCT AAAGAA CTA AG | 222 | [2] |
|  | R: GGG ACC AAC ATA ACC TAA |  |  |

**Primers for the Detection of Antibiotic Resistance Genes in Gram-negative Bacterial Isolates**

| **Gene** | **Primer Sequence (5'-3')** | **Size (bp)** | **Reference** |
| --- | --- | --- | --- |
| *blaSHV* | F: CGCCTGTGTATTATCTCCCT | 293 | [3] |
|  | R: CGAGTAGTCCACCAGATCCT |  |  |
| *aac (3)-II* | F: ATATCGCGATGCATACGCGG | 877 | [4] |
|  | R: GACGGCCTCTAACCGGAAGG |  |  |
| *tetA* | F: GCTACATCCTGCTTGCCTTC | 209 | [5] |
|  | R: ATAGATCGCCGTGAAGAGG |  |  |
| *dfr1* | F: CGAAGAATGGAGTTATCGGG | 372 | [5] |
|  | R: TGCTGGGGATTTCAGGAAAG |  |  |

1. Matsuda K, Tsuji H, Asahara T, Kado Y, Nomoto K. Sensitive Quantitative Detection of Commensal Bacteria by rRNA-Targeted Reverse Transcription-PCR. *Appl Environ Microbiol*. 2007;73(1):32-39.

2. Tarazi YH, Almajali AM, Kheer Ababneh MM, Ahmed HS, Jaran AS. Molecular study on methicillin-resistant Staphylococcus aureus strains isolated from dogs and associated personnel in Jordan. *Asian Pacific Journal of Tropical Biomedicine*. 2015;5(11):902-908.

3. Sharif M, Mirnejad R, Amirmozafari N. Molecular identification of TEM and SHV extended spectrum $\beta$-lactamase in clinical isolates of Acinetobacter baumannii from Tehran hospitals. *The Journal of Genes, Microbes and Immunity*. 2014;2014:1-9.

4. Hu X, Xu B, Yang Y, et al. A high throughput multiplex PCR assay for simultaneous detection of seven aminoglycoside-resistance genes in Enterobacteriaceae. *BMC Microbiol*. 2013;13(1):58.

5. Adesiyan IM, Bisi-Johnson MA, Ogunfowokan AO, Okoh AI. Incidence and antimicrobial susceptibility fingerprints of Plesiomonas shigelliodes isolates in water samples collected from some freshwater resources in Southwest Nigeria. *Science of The Total Environment*. 2019;665:632-640.
