# Supplementary material for: Molecular characterization of antibiotic resistance in bacteria from daycare centres in Ile-Ife, Nigeria
Source: JAC Antimicrob Resist. 2024 Dec 30;7(1):dlae213. doi: 10.1093/jacamr/dlae213 (PMC11683008; doi:10.1093/jacamr/dlae213)
Supplement: dlae213_Supplementary_Data [file dlae213_supplementary_data.zip › Supplementary File 3.docx]

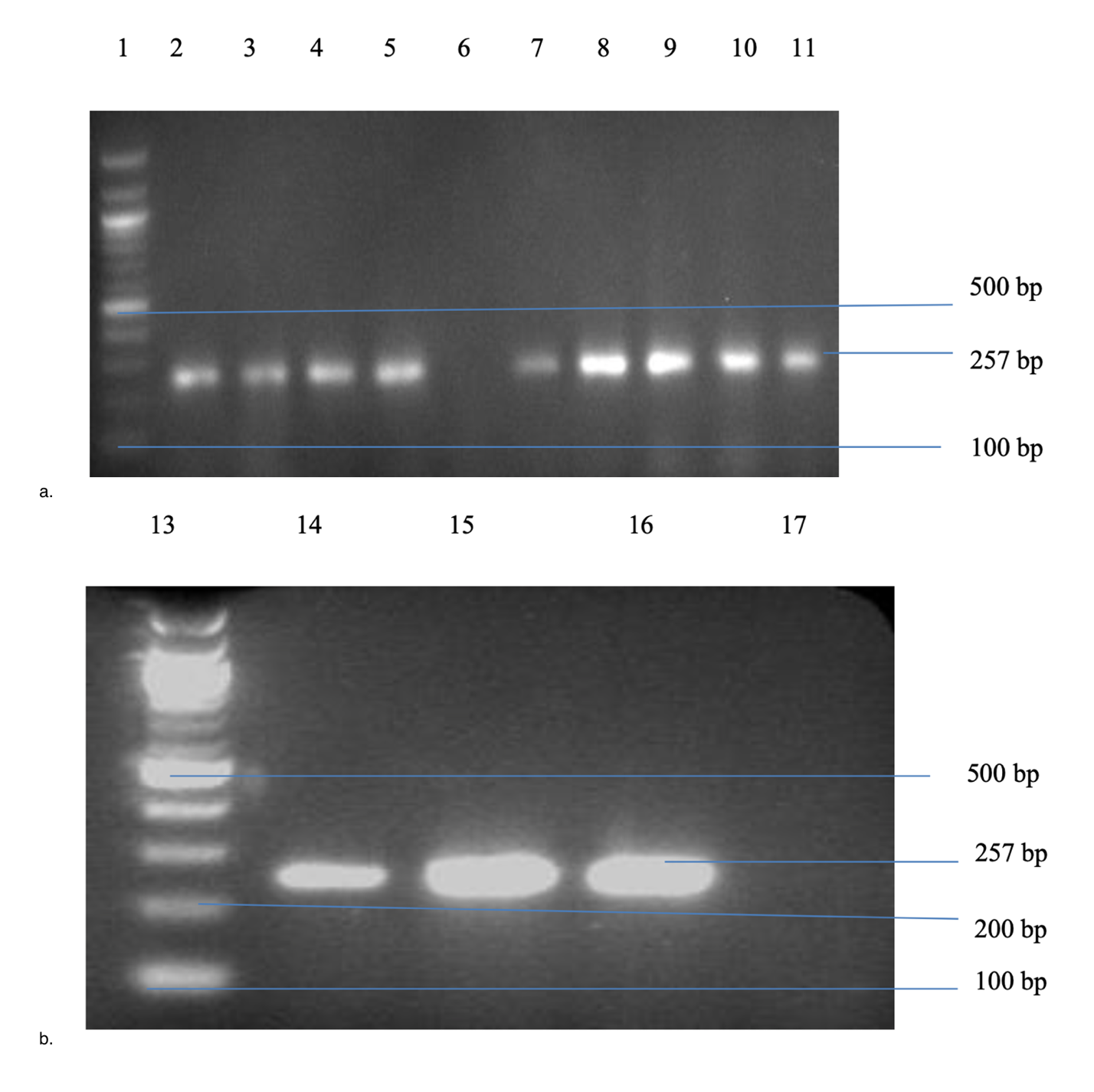


Agarose gel electrophoresis for the amplification product showing the 16srRNA in *S. aureus* (257 bp) in the selected isolates

*a. Lane 1: 100 bp Ladder; Lanes 2-5 and Lanes 7-12 – S. aureus. b. Lane 1- 100 bp Ladder; Lanes 14 - 16- S. aureus*


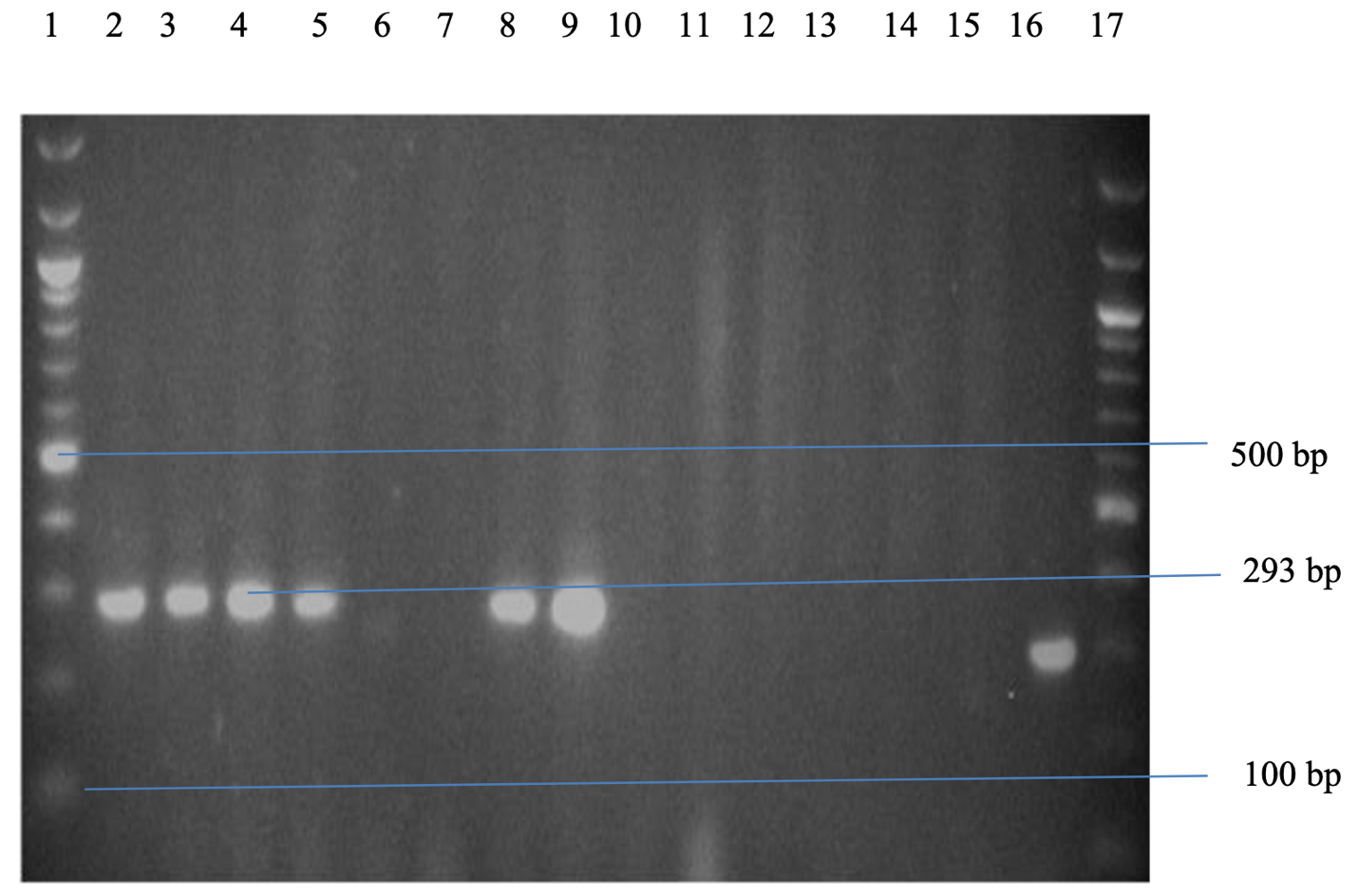


Agarose gel electrophoresis of the amplification product coding *blaSHV* gene (293 bp) in the selected isolates
*Lane 1: 100 bp Ladder; Lane 2: Citrobacter youngae, Lane 3-5: Klebsiella oxtyoca and Lane 8-9: K. pneumoniae. Lane 11: K. oxtyoca (control). Lane 16: K. pneumoniae*


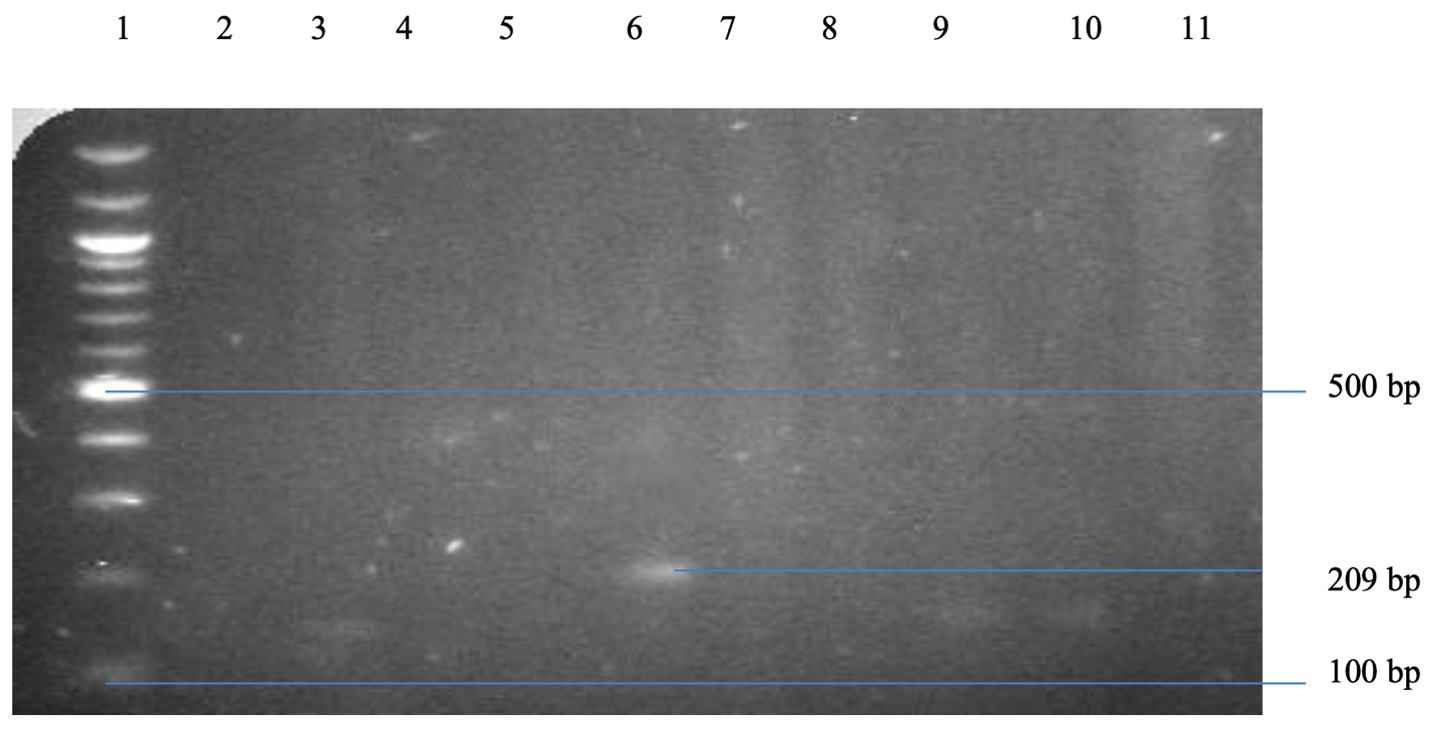


Agarose gel electrophoresis of the amplification product coding *tetA* gene (209 bp) in the selected isolates.
*Lane 1: 100 bp Ladder; Lane 6: E. coli*


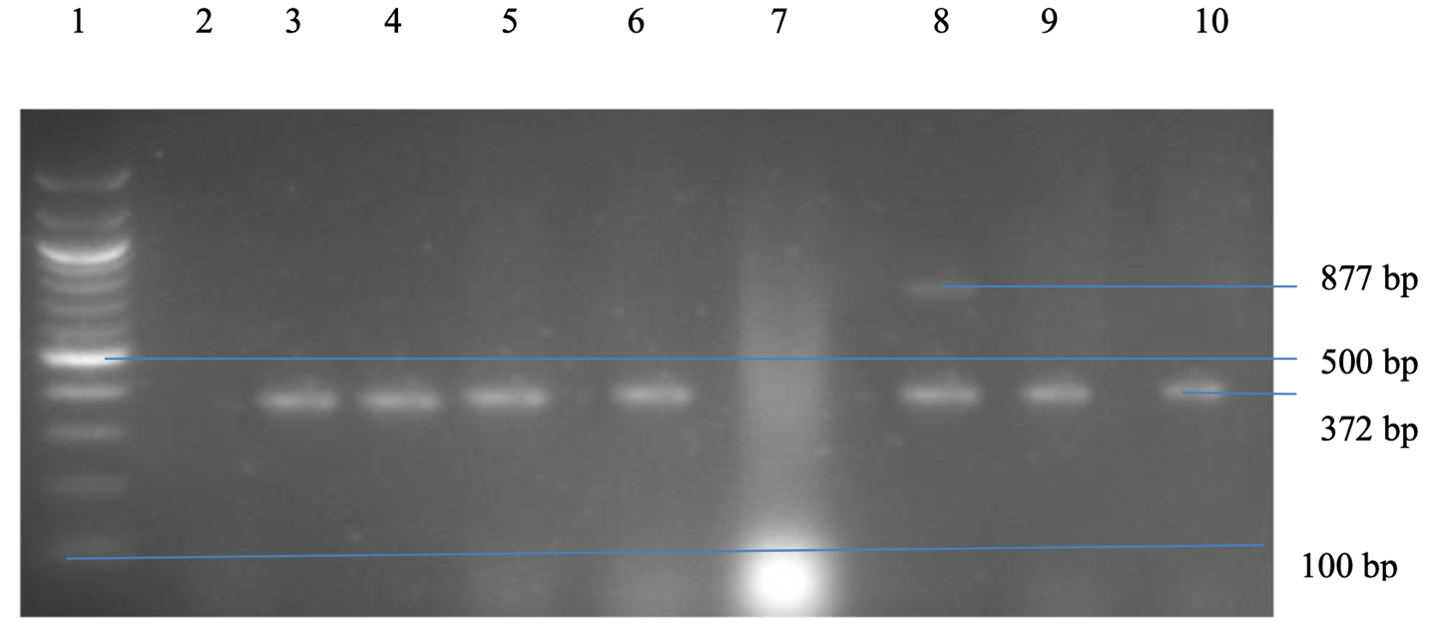


Multiplex PCR amplification products by agarose gel electrophoresis for the *dfr1* gene (372 bp) and *acc (3)-II* (877 bp) in the selected isolates.
*Lane 1: 100 bp Ladder; Lane 3- C. youngae, Lane 4: K. oxytoca, Lane 5-6- K. pneumoniae, Lane 8: E. coli. Lane 9-10: Providencia rettgeri*


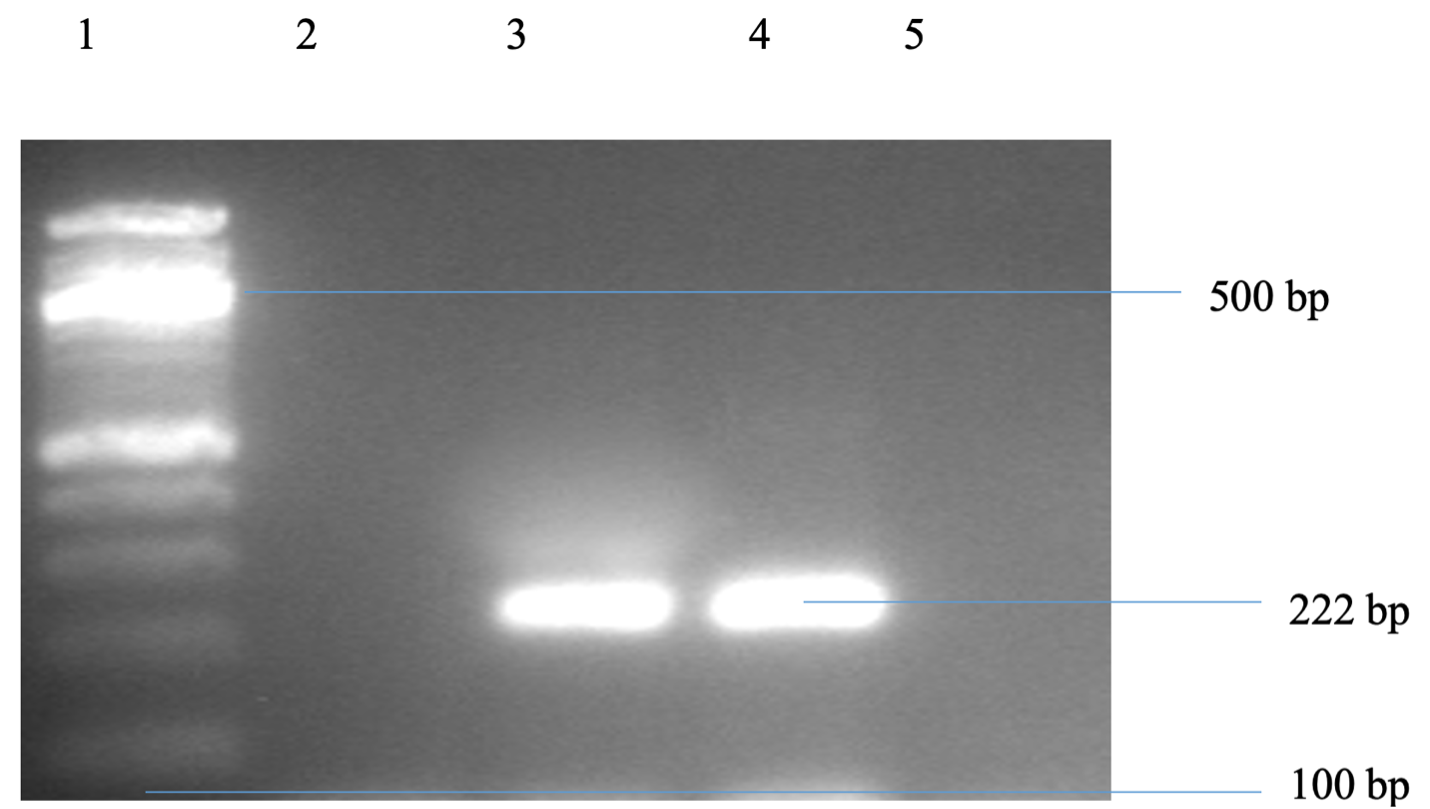


Agarose gel electrophoresis of the amplification product coding *MecA* gene (222 bp) in the selected *S. aureus* isolates.
*Lane 1: 100 bp; Ladder; Lane 3-4 – S. aureus*
